# Orthogonal Hydroxyl Functionalization of cGAMP Confers Metabolic Stability and Enables Antibody Conjugation

**DOI:** 10.1021/acscentsci.3c01122

**Published:** 2023-11-15

**Authors:** Yong Lu, Lin You, Liping Li, Jessica A. Kilgore, Shun Liu, Xiaoyu Wang, Yuanwei Dai, Qi Wei, Heping Shi, Lei Han, Lijun Sun, Zhijian J. Chen, Xuewu Zhang, Noelle S. Williams, Chuo Chen

**Affiliations:** ^†^Department of Biochemistry, ^‡^Pharmacology, and ^§^Molecular Biology UT Southwestern Medical Center 5323 Harry Hines Boulevard, Dallas, Texas 75390, United States

## Abstract

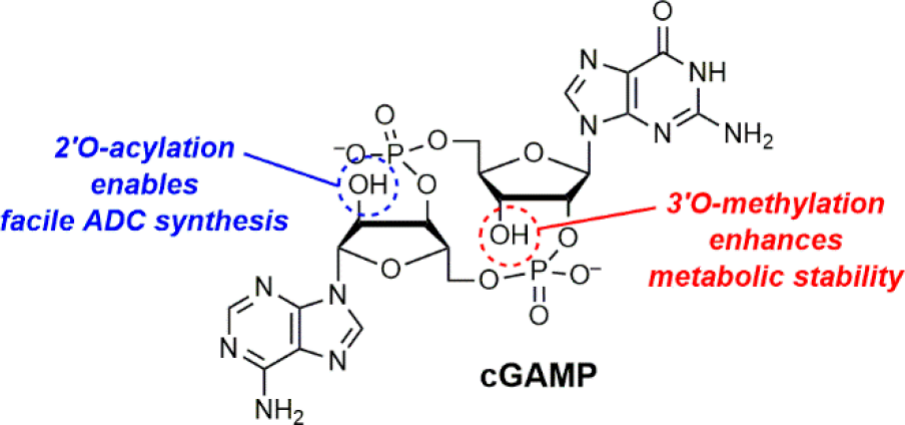

cGAMP is a signaling molecule produced by the cGAS–DNA complex to establish antimicrobial and antitumor immunity through STING. Whereas STING activation holds potential as a new strategy to treat cancer, cGAMP is generally considered unsuitable for in vivo use because of the rapid cleavage of its phosphodiester linkages and the limited cellular uptake under physiological conditions. Consequently, phosphorothioation and fluorination are commonly used to improve the metabolic stability and permeability of cGAMP and its synthetic analogues. We now show that methylation of the 3′-hydroxyl group of cGAMP also confers metabolic stability and that acylation of the 2′-hydroxyl group can be achieved directly and selectively to enable receptor-mediated intracellular delivery. Unlike phosphorothioation and fluorination, these modifications do not create a new stereogenic center and do not require laborious building block synthesis. As such, orthogonal hydroxyl functionalization is a simple solution to issues associated with the in vivo use of cGAMP.

## Abstract 2

3′*O*-Methylation protects cGAMP from ENPP1 hydrolysis, and 2′*O*-acylation enables easy conjugation to an antibody.

## Introduction

**Figure 1:**
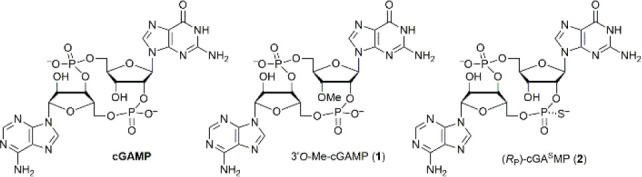
Chemical structures of cGAMP, 3′*O*-Me-cGAMP (**1**), and (*R*_P_)-cGA^S^MP (**2**).

The detection of DNA in the cytoplasm provides a cue for cells to launch immune responses to pathogens and cancer. A key step of this process is the production of 2′,3′-cGAMP (cGAMP hereafter, Figure 1)^1−3^ to relay the DNA danger signal for interferon induction. cGAMP is a cyclic dinucleotide (CDN) generated from ATP and GTP by cGAS (cyclic-GMP-AMP synthase) upon DNA complexation.^4^ cGAMP binds to and activates the adaptor protein STING (stimulator of interferon genes), promoting the assembly of a signalosome to support downstream innate immune signaling.^5^ This event is vital to establishing anticancer immunity^6^ and is required for responding to the immune checkpoint blockade treatment of cancer.^7^ Consequently, STING activation has been vigorously pursued as a new immunotherapy for cancer.^8^

Previously, we and others^9−12^ have found that mammalian cGAMP is a hybrid CDN^1^ and contains mixed phosphodiester linkages.^2^ It adopts a closed conformation that can transition easily into the STING-bound conformation.^13^ However, phosphodiesterase ENPP1 (ectonucleotide pyrophosphatase/phosphodiesterase 1) degrades cGAMP rapidly^14−16^ and limits its therapeutic utility. Consequently, virtually all CDN-type STING agonists in clinical development contain a phosphorothioate modification.^17^ Despite being effective, this strategy introduces a new *P*-chiral center that can be challenging to set.^18−20^ We show herein that 3′*O*-methylation protects cGAMP against enzymatic digestion in vitro and improves the overall exposure in vivo more effectively than phosphorothioation. Additionally, selective 2′*O*-acylation of cGAMP can be achieved to enable convenient antibody–drug conjugate (ADC) synthesis.^21−23^ Loading 3′*O*-Me-cGAMP (**1**) onto an anti-PD-L1 antibody allows for its targeted delivery into cells in a PD-L1-dependent manner. Taken together, this work demonstrates that the therapeutic utility of cGAMP can be improved easily by orthogonal hydroxyl functionalization.

## Results and Discussion

**Figure 2:**
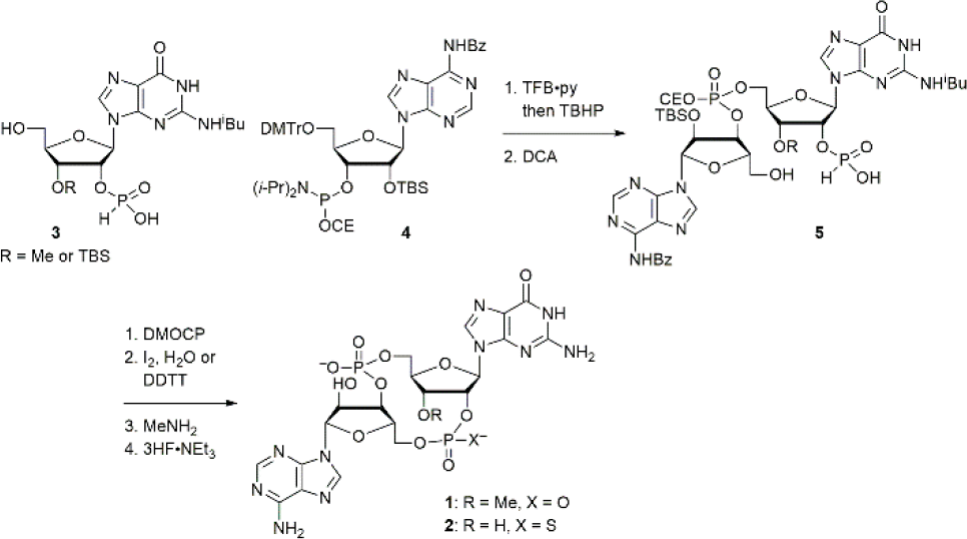
Synthesis of **1** and **2**. Both CDNs can be easily prepared by conventional P(III) chemistry.

**Figure 3:**
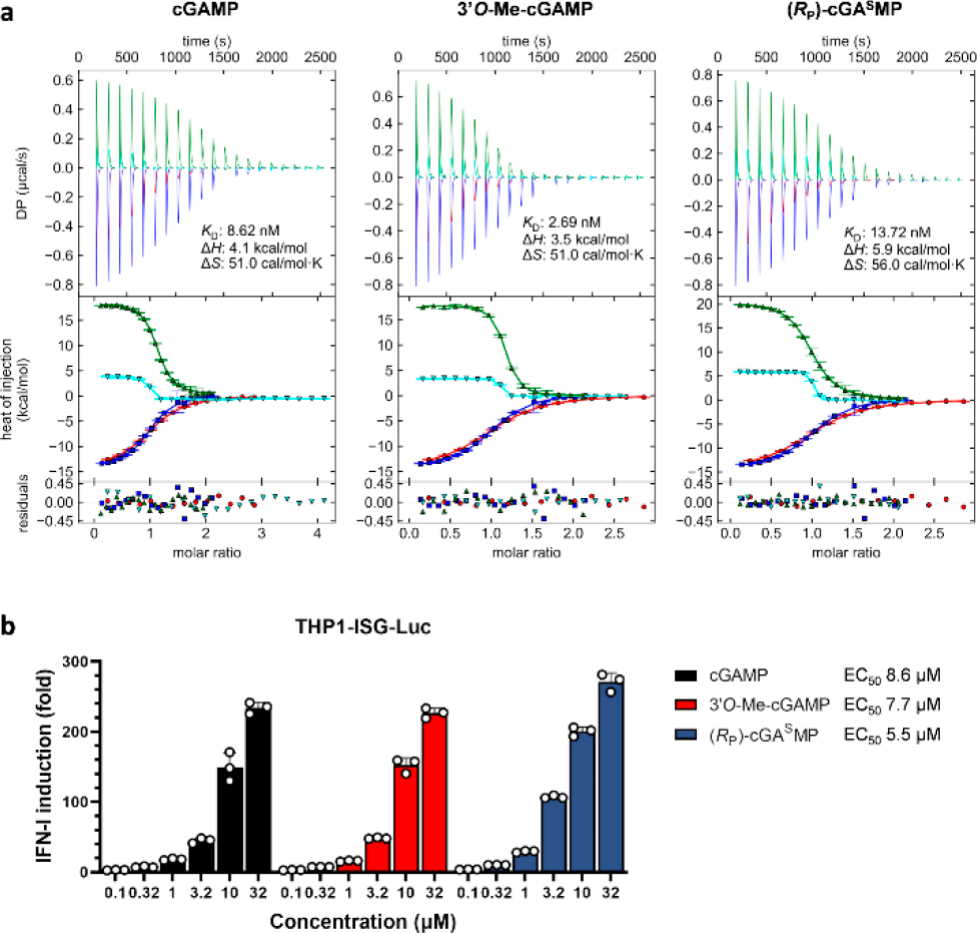
Affinities and activities of cGAMP, **1**, and **2**. (a) The original titration traces (top) and integrated data (bottom) of the ITC experiments. 15 μM (red) and 30 μM (blue) apo-STING dimer solutions were titrated by c-di-GMP (350 μM), respectively. Apo-STING dimer (cyan) and c-di-GMP-bound STING dimer (green) were titrated by cGAMP, **1**, or **2**, respectively. (b) The abilities of these CDNs to induce interferons were assessed in THP-1 cells using a Lucia luciferase reporter controlled by an IRF-inducible promoter.

Phosphorothioation and *O*-methylation are two common strategies to improve the stability of oligonucleotides that contain canonical (3′ → 5′)-phosphodiester linkages.^24,25^ Protecting the noncanonical (2′ → 5′)-phosphodiester linkage of cGAMP by phosphorothioation (i.e., **2**) is also known to hinder ENPP1 digestion,^14^ but the impact of *O*-methylation on the stability of cGAMP has not been reported. As protecting the noncanonical phosphodiester linkage with methylation would involve modification of the 3′*O*- instead of the common 2′*O*-hydroxyl group of the ribonucleotide unit, it is not clear if such modification would confer the same protection. To address this question, we prepared **1** using the conventional phosphoramidite and *H*-phosphonate chemistry (Figure 2).^26,27^ Briefly, coupling of **3** (R = Me)^28^ and **4** using pyridinium tetrafluoroborate as the promoter^29,30^ followed by *P*-oxidation and deprotection gave dinucleotide **5**. Subsequent cyclization, oxidation, and deprotection provided **1**. For comparison, we also prepared **2** by the same method using DDTT (3-((dimethylamino-methylidene)amino)-3*H*-1,2,4-dithiazole-3-thione or sulfurizing reagent II)^31^ as the oxidant for the second P(III) oxidation. Consistent with docking prediction (Supporting Information Figure S1), ITC (isothermal titration calorimetry) experiments showed that **1** retains affinity to the ligand-binding domain of the “wild-type” STING (R232) (Figure 3a and Supporting Information Figure S2) and the ability to activate HAQ STING (H71, A230, Q293) in THP-1 cells (Figure 3b).

**Figure 4:**
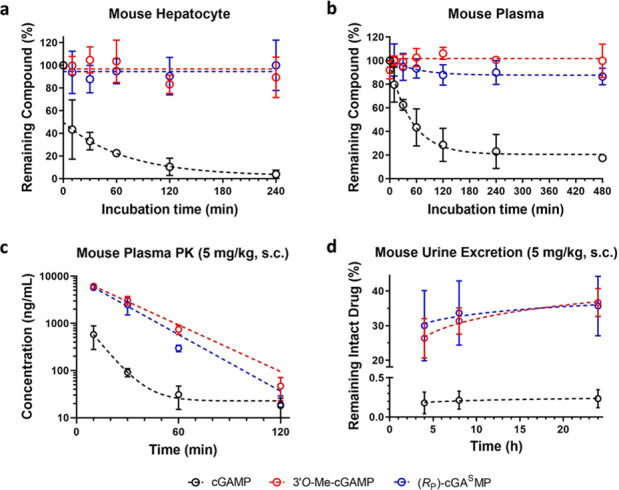
In vitro metabolic stability and in vivo pharmacokinetic properties of cGAMP, **1** and **2**. (a and b) The concentrations of CDNs after incubating with (a) mouse hepatocytes or (b) mouse plasma. (c and d) The concentrations of CDNs in (c) blood and (d) urine after subcutaneous administration (5 mg/kg).

ENPP1 is an ecto-enzyme highly expressed on the surface of plasma cells and liver cells.^32^ Intracellular processing of mouse ENPP1 at Lys85 (Lys103 in human ENPP1) also leads to a soluble form that can be secreted into the serum.^33^ Consistently, we found that cGAMP was stable to mouse liver microsomes (Supporting Information Figure S3) but was degraded rapidly by mouse hepatocytes (Figure 4a). By contrast, **1** and **2** remained intact 4 h after incubating with mouse liver microsomes or hepatocytes. Similarly, cGAMP was lost nearly completely whereas **1** and the majority of **2** could be recovered from the mouse plasma after an 8 h incubation (Figure 4b). Recent crystallographic studies have revealed that the Thr238 residue of mENPP1 (Thr256 in hENPP1) is responsible for the hydrolysis of cGAMP.^16^ However, based on this model, introducing a 3′*O*-methyl group would not block the nucleophilic attack of Thr238 at the phosphorus center (Supporting Information Figure S4). The mechanism by which 3′O-methylation prevents ENPP1 digestion is unclear. But because cGAMP needs to adopt an open conformation for ENPP1 binding, it is likely that 3′*O*-methylation hinders the requisite closed-to-open conformational change^13^ (Supporting Information Figure S5) and thus provides protection toward ENPP1 digestion.

**Figure 5:**
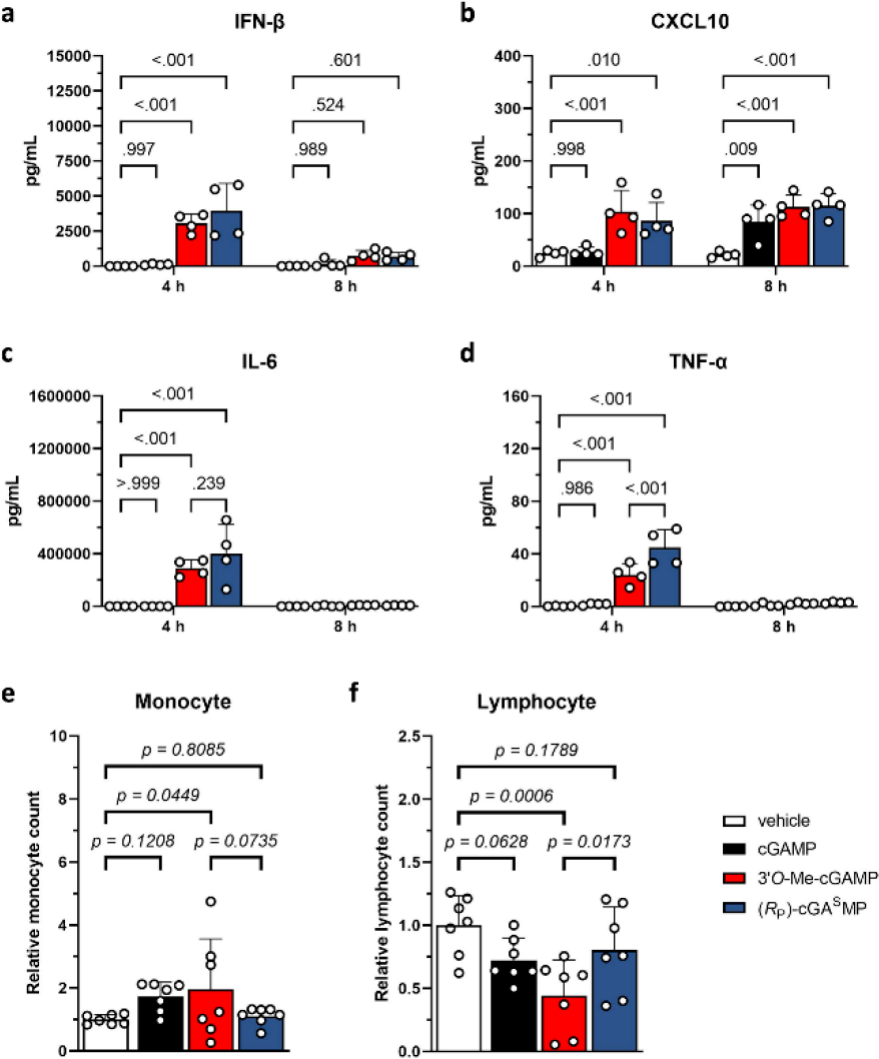
In vivo pharmacodynamic properties of cGAMP, **1**, and **2** in mice. The amount of (a) IFN-β, (b) CXCL10, (c) IL-6, and (d) TNF-α in blood 4 and 8 h after subcutaneous administration of the CDN (5 mg/kg). The relative amount of (e) monocyte and (f) lymphocyte in blood at the 4 h time point.

We next compared the pharmacokinetic properties of **1** and **2** with those of cGAMP in mice. When administrated subcutaneously, all these molecules entered systemic circulation rapidly (Figure 4c). The drug concentration reached maximum in plasma within 10 min and then dropped quickly in all cases. However, the clearance rate of **1** and **2** were significantly slower than that of cGAMP, leading to increased drug concentrations in the plasma. Additionally, unlike cGAMP that was removed from blood with a complex mechanism, **1** and **2** were eliminated with simple first-order kinetics. We also recovered a significant amount of **1** and **2** from the urine (Figure 4d), suggesting that urinary excretion is a major elimination pathway for these stable cGAMP analogues. The recovery of all compounds from the feces was below the detection limit of our LC/MS method (10 ng/mL). These observations are consistent with the molecular characteristic of CDNs that carry two negative charges under physiological conditions. We have also analyzed the blood cytokine levels 4 and 8 h after administration and found that the levels of IFN-β, CXCL10, IL-6, and TNF-α were significantly higher with **1** and **2** (Figure 5a–d). Interestingly, **1** induced less TNF-α than **2** (Figure 5d) and more notable changes in the number of monocytes and lymphocytes than cGAMP and **2** (Figure 5e,f). Taken together, **1** is a simple modification of cGAMP that provides better immune responses than cGAMP meanwhile posting a lower risk of systemic inflammation than **2**.

**Figure 6:**
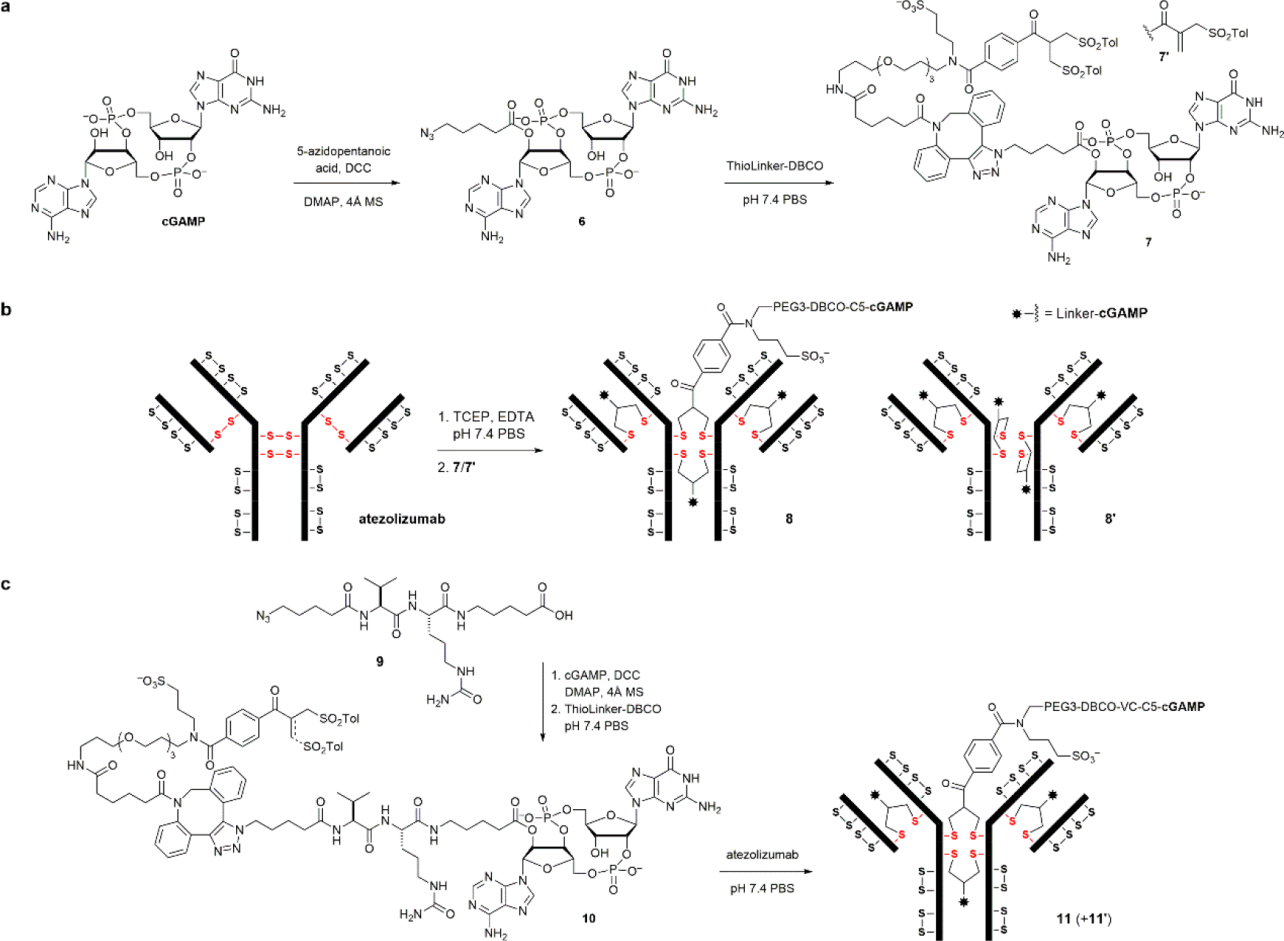
Synthetic route to the cGAMP–atezolizumab conjugates. (a) Functionalization of cGAMP at the 2′O-position can be achieved by DCC coupling. (b) cGAMP can then be load onto the antibody by ETAC. (c) Synthesis of a cGAMP–ADC with a cathepsin-cleavable linker.

Whereas the above results indicate that systemic administration is a viable option, CDNs are usually administered by intratumoral injection to ensure local activation of STING. Several transporters are known to carry cGAMP across the cell membrane.^34−39^ We incubated THP-1 cells with 10 μM of cGAMP and found that the intracellular level of cGAMP was stably maintained at ∼1.5 μM from 2 to 24 h (Supporting Information Figure S6), indicating a tight regulation of its uptake. To assist the delivery and retention of CDN at the tumor site, we investigated methods to conjugate cGAMP to a tumor-targeting agent. Taking advantage of the overexpression of PD-L1 on the surface of tumor cells and tumor-infiltrating immune cells, we envisioned that loading cGAMP onto atezolizumab, a clinically used anti-PD-L1 antibody, would allow for enhancing anticancer immunity and blocking the downstream PD-1/PD-L1 signaling^40−42^ at the same time. We chose to focus on the disulfide rebridging method^43−49^ because it can provide a better control of the ADC homogeneity and drug–antibody ratio (DAR) than the conventional cysteine conjugation method without reengineering the antibody.^50^ To this end, we found that the 2′-hydroxyl group can be selectively functionalized by DCC (dicyclohexyl carbodiimide) coupling to facilitate linker installation. A slow addition of DCC and 5-azidopentanoic acid to cGAMP led to the formation of **6** (Figure 6a). We then explored the equilibrium transfer alkylating cross-link (ETAC) method^51^ for antibody conjugation. Reacting **6** with the commercially available ETAC reagent ThioLinker-DBCO gave an equilibrium mixture of bis-sulfone **7** and allylsulfone **7′** (the active form for bioconjugation) as well as their triazole regioisomers. Meanwhile, the antibody was reduced by TCEP (tris(2-carboxyethyl)phosphine)^52^ to cleave the four interchain disulfides (Figure 6b).^53,54^ The released sulfide pairs were then rebridged by **7**/**7′** at pH 7.4 wherein the inactive form **7** was converted to the active form **7′** in situ. The near-neutral conditions were used to minimize the undesired lysine conjugation that would occur under more basic conditions.^55^

**Figure 7:**
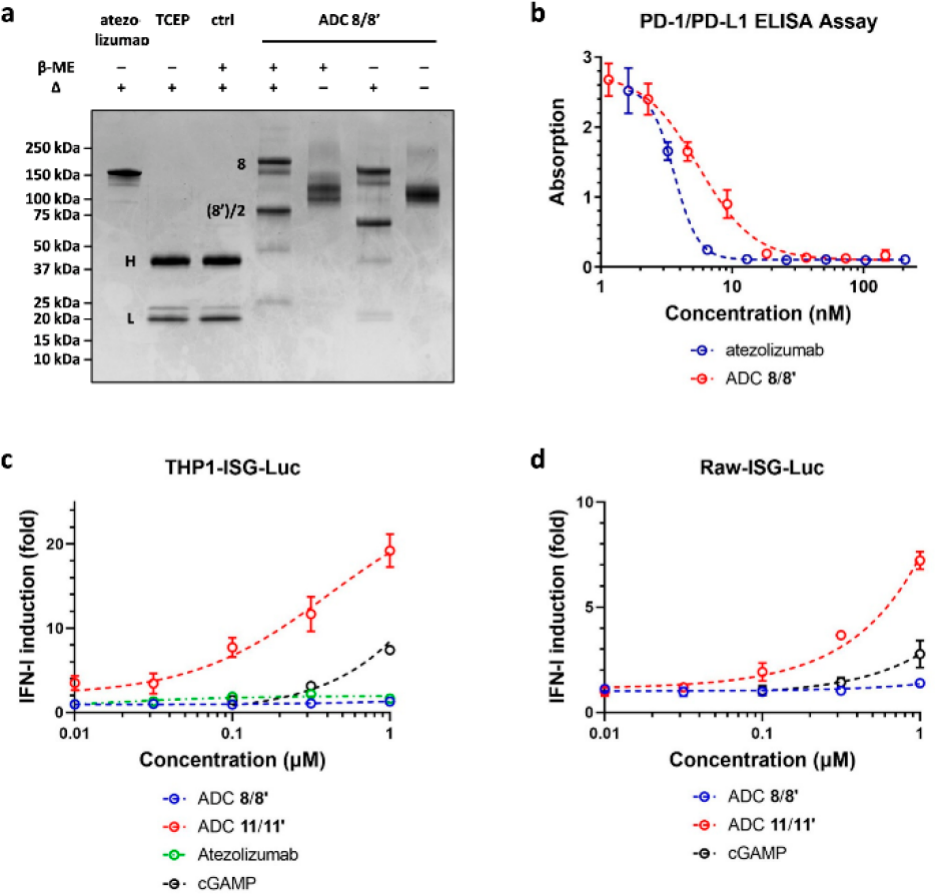
Characterization of cGAMP–ADCs **8**/**8′** and **11**/**11′**. (a) SDS-PAGE analysis of **8**/**8′** under reducing/nonreducing and denaturing/native conditions. (b) Validation of the binding of **8**/**8′** to PD-L1 by ELISA. ADC **11**/**11′** is more potent than cGAMP in inducing interferons in (c) THP-1 and (d) Raw264.7 cells while **8**/**8′** failed to active both human and mouse STING.

**Figure 8:**
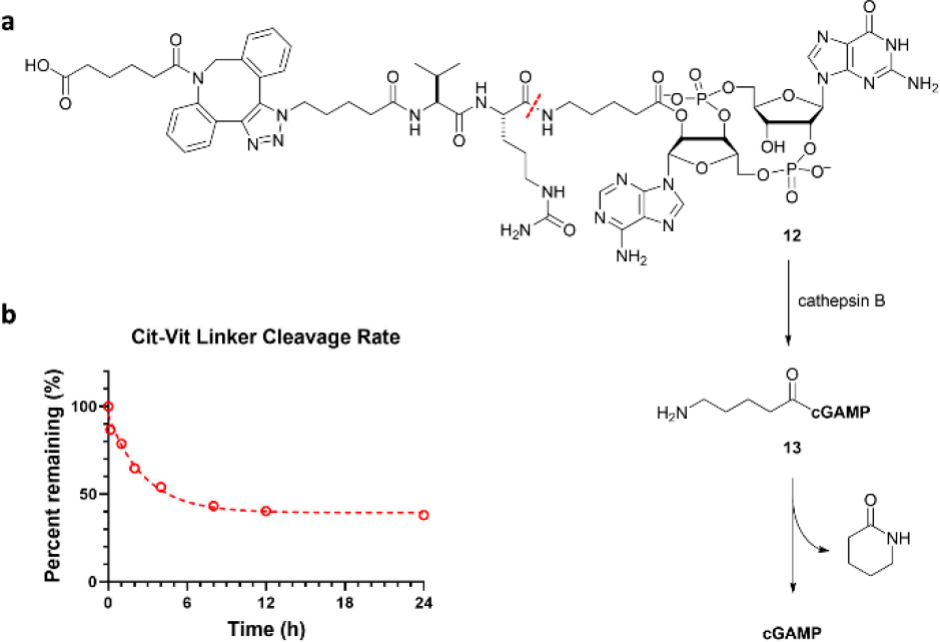
Release rate and the stability of cGAMP. (a) The mechanism of the payload release of the model substrate **12**. (b) **12** was consumed upon incubation with cathepsin B in a time-dependent manner.

The resulting ADC has a desired DAR value of 4.2 based on its UV profile (Supporting Information Figure S7). However, SDS-PAGE and intact MS analyses showed that partial scrambling of the interheavy chain disulfides occurred to give a stable dimer of the conjugated half-body **8′** in addition to the rebridged **8** (Figures 6b and 7a, and Supporting Information Figure S8).^56^ The binding of **8**/**8′** toward human PD-L1 was tested by ELISA (enzyme-linked immunosorbent assay). ADC **8**/**8′** competed with biotinylated PD-1 for binding to PD-L1 with an IC_50_ value comparable to that of the unmodified atezolizumab (5.5 nM vs 3.6 nM) (Figure 7b). Thus, functionalization of atezolizumab by ETAC did not affect its affinity toward PD-L1 significantly. The observed change of the Hill coefficient from −4.8 to −2.1 is likely due to the heterogeneity of the ADC. Alternatively, it may suggest that loading cGAMP onto the atezolizumab affected its binding avidity. Surprisingly, despite high affinity, ADC **8**/**8′** failed to activate STING in THP-1 cells (Figure 7c). We reasoned that the lack of activity was due to ineffective cGAMP release. Because **8**/**8′** bears a stable linker, hydrolysis of the hindered 2′*O*-acyl group is needed to release the payload after internalization. To address this issue, we introduced to the linker a Val-Cit dipeptide unit that can be cleaved by cathepsin in a location- and carrier-independent manner.^23,57,58^ DCC coupling of **9** and cGAMP followed by reacting with ThioLinker-DBCO gave **10** that could also be loaded onto atezolizumab smoothly to give ADC **11**/**11′** (DAR 3.9) (Figure 6c). Pleasingly, **11**/**11′** induced interferons in THP-1 cells in a dose-dependent manner and was more potent than cGAMP (Figure 7c). It also activated mouse STING in Raw 264.7 cells more effectively than cGAMP (Figure 7d). This result is consistent with the notion that atezolizumab is a humanized antibody active toward both human and mouse PD-L1. To confirm that **11**/**11′** functioned through the designed mode of action, we used **12** as the model substrate and monitored the release of cGAMP through **13** in vitro (Figure 8a). Indeed, **12** was consumed over time in the presence of cathepsin B (Figure 8b).

**Figure 9:**
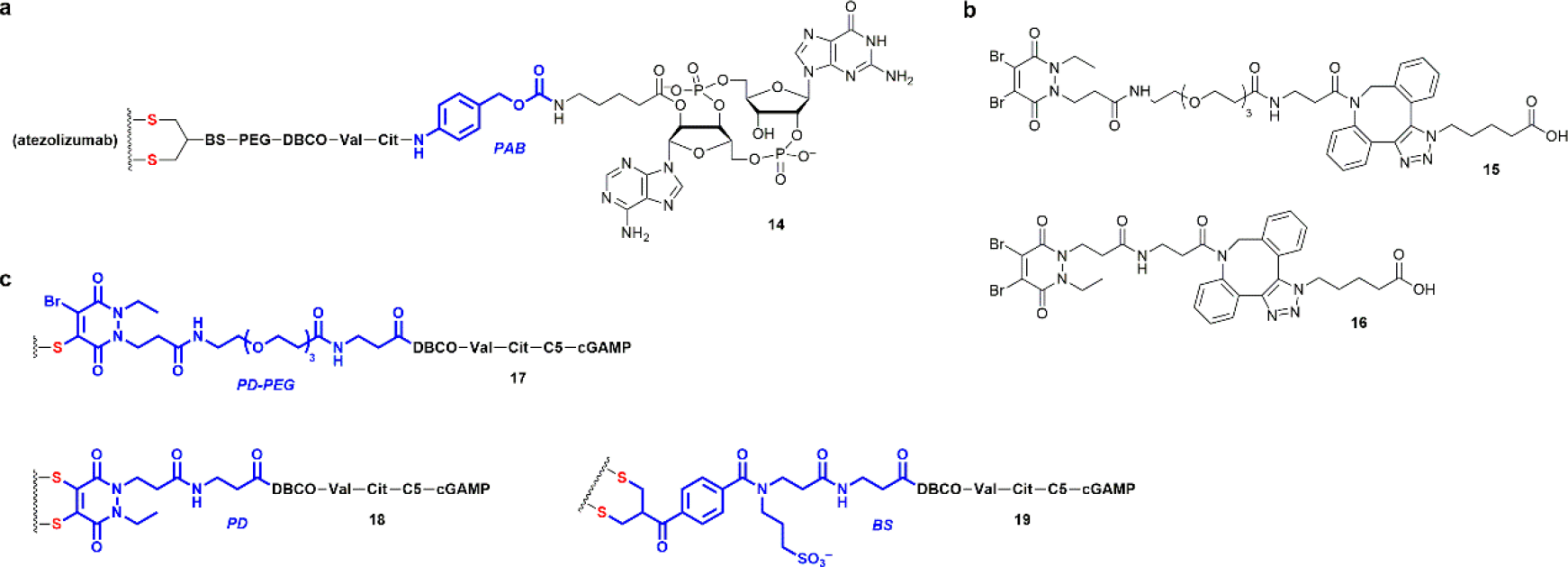
Exploration of the ADC linker chemistry. (a) The structure of the bis-sulfone-derived ADC **14** bearing a PAB moiety. (b) The structures of the PEGylated and non-PEGylated diBrPD linkers **15** and **16**. (c) The structures of the diBrPD-derived ADCs **17** and **18**, and that of the bis-sulfone-derived ADC **19** corresponding to **18**.

Because *p*-aminobenzyl (PAB) group is a self-immolative spacer^59^ frequently used in conjunction with Val-Cit to facilitate payload release, we asked if incorporating PAB into **11**/**11′** would facilitate the release of cGAMP (Figure 9a). Interestingly, whereas ADC **14** could also activate STING, it is less potent than **11**/**11′** in inducing interferons in THP-1 cells (Supporting Information Figure S9a). The PAB group should thus be used judiciously in ADC design. We next tested if disulfide rebridging by the dibromopyridazinedione (diBrPD) method^60−62^ would provide improved activity. Unlike bis-sulfone, diBrPD is compatible with TCEP, which allows for disulfide reduction and cysteine conjugation to be performed in one pot. However, the conjugation needed to be carried out in slightly basic conditions to grant cysteine sufficient reactivity toward diBrPD. Indeed, **15** (Figure 9b) reacted slowly with 1-butanethiol over 2 d at pH 9 while no reaction occurred at pH 7.4. Interestingly, the non-PEGylated **16** was more reactive and could be fully consumed within 5 h when incubated with 1-butanethiol at pH 9. Consistently, the diBrPD–PEG–Val–Cit–cGAMP reacted with atezolizumab sluggishly to give ADC **17** (Figure 9c) with a DAR value of ∼10 without rebridging the disulfides. Meanwhile, direct conjugation of atezolizumab with diBrPD–Val–Cit–cGAMP was also difficult. We thus functionalized atezolizumab with diBrPD–DBCO first and then reacted it with Val–Cit–cGAMP to generate ADC **18**. For comparison, we also prepared the corresponding bis-sulfone ADC **19**. Interestingly, excluding the PEG group prevented bis-sulfone from in situ elimination of a sulfone unit under neutral conditions to generate the active allylsulfone for conjugation. Thus, prior activation of the bis-sulfone by base treatment^63^ was needed to enable the synthesis of ADC **19**. Both **18** and **19** were less potent than **11** in activating STING in THP-1 cells (Supporting Information Figure S9b). These results suggest that the hydrophobicity of the linker can have a significant impact on the rate of cathepsin digestion and the efficiency of cysteine conjugation. Of note, we have not been able to use the diBrPD method to generate a rebridged cGAMP–ADC with a PEG–Val–Cit linker.

**Figure 10:**
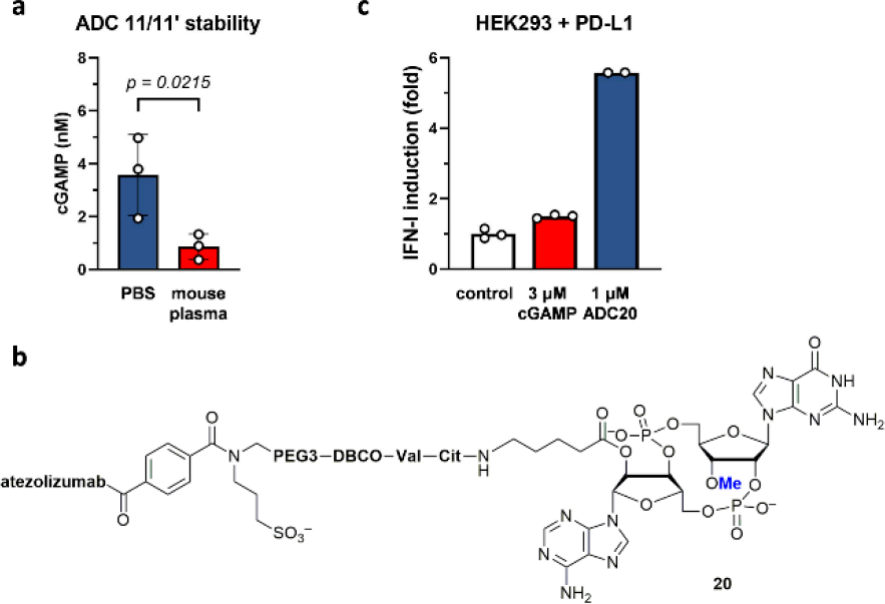
Orthogonal functionalization of cGAMP allows for enhanced STING activation by ADC in an antigen-dependent manner. (a) The cGAMP on ACD **11**/**11′** (DAR 3.9, 1 nM) was partially hydrolyzed after incubating in mouse plasma for 4 h. The amount of intact cGAMP was determined by ELISA after releasing from **11**/**11′** by cathepsin B in the presence of STF-1084. (b) The structure of 3′*O*-Me-cGAMP–atezolizumab conjugate **20**. (c) ADC **20** induced interferons more effectively in HEK293 cells expressing PD-L1 than control, but cGAMP was insensitive to PD-L1 overexpression.

We next determined whether conjugating cGAMP to an antibody by 2′*O*-acylation is sufficient to protect it from hydrolysis in vivo. Unfortunately, the amount of cGAMP released from ADC **11**/**11′** by cathepsin in the presence of STF-1084,^64^ an ENPP1 inhibitor, reduced signficantly after incubating it in mouse plasma for 5 h (Figure 10a). Nonetheless, because the protection of cGAMP by methylation and the acylation of cGAMP for ADC synthesis involve orthogonal functionalization of its hydroxyl groups, we envisioned that these two modifications could be used together to provide enhanced and durable STING activation. Indeed, ADC **20** (Figure 10b) could be prepared smoothly from **1** by the DCC and bis-sulfone chemistry. To demonstrate that **20** improves the intracellular delivery of **1** to activate STING in a PD-L1-dependent manner, we transfected PD-L1 plasmid into HEK293 cells (Supporting Information Figure S10) and found that PD-L1 overexpression significantly enhanced the activation of STING by **20** but not cGAMP (Figure 10c).

## Conclusion

The therapeutic potential of cGAMP is limited by its rapid degradation and moderate uptake in vivo. Whereas it is generally believed that systemic delivery of CDN is not a viable option, we have found that cGAMP and its stable analogues could enter circulation readily upon subcutaneous injection. However, cGAMP is rapidly eliminated by hydrolysis and stable CDNs by urinary excretion. Additionally, the transporter-mediated internalization of cGAMP is a regulated process, making the intracellular concentration of cGAMP maintained at a level significantly lower than that of the local environment. Thus, the efficiency of the commonly used intratumoral injection of CDNs may be limited by rapid absorption and subsequent elimination through circulation.

Phosphorothioation has been used widely to improve the metabolic stability of CDNs. However, we have found that 3′*O*-methylation is also effective in protecting cGAMP. This simple modification does not introduce a new stereogenic center and appears to provide a better safety profile than phosphorothioation. Meanwhile, 2′*O*-acylation can be achieved readily to enable ADC synthesis, and ADC can help deliver CDN directly into immune or cancer cells at the tumor site to induce anticancer immunity without generating a high extracellular concentration of the free drug. Thus, orthogonal hydroxyl functionalization of cGAMP may provide an ADC capable of activating STING more safely and effectively. Previously, we reported a STING-targeting ADC that requires reengineering of the warhead through lengthy synthesis to enable conjugation.^65^ The current approach of 2′O-acylation is more flexible and can serve as a general strategy to produce ADCs from cGAMP and various CDNs currently under clinical or preclinical development. However, because stable STING agonists would elicit systemic inflammation if released prematurely or leaked from the tumor site, caution should be used in applying STING ADCs with a slow systemic clearance of their warheads.^66^ Effort toward addressing the safe use of STING agonists in vivo through ADC optimization is underway.

## Supporting Information Available

Experimental procedures (PDF)
