# Supplementary material for: Orthogonal Hydroxyl Functionalization of cGAMP Confers Metabolic Stability and Enables Antibody Conjugation
Source: ACS Cent Sci. 2023 Nov 15;9(12):2298–305. doi: 10.1021/acscentsci.3c01122 (PMC10755847; doi:10.1021/acscentsci.3c01122)
Supplement: Supplementary file 1 — oc3c01122_si_001.pdf [file oc3c01122_si_001.pdf]

Supporting Information

Orthogonal Hydroxyl Functionalization of cGAMP Confers Metabolic Stability and Enables Antibody Conjugation

Yong Lu,[1] Lin You,[1] Liping Li,[1] Jessica A. Kilgore,[1] Shun Liu,[2] Xiaoyu Wang,[1] Yuanwei Dai,[1] Qi Wei,[1] Heping Shi,[1] Lei Han,[1] Lijun Sun,[3] Zhijian J. Chen,[3] Xuewu Zhang,[2] Noelle S. Williams[1] and Chuo Chen[1,*]

[1]Department of Biochemistry, [2]Pharmacology, and [3]Molecular Biology

UT Southwestern Medical Center

5323 Harry Hines Boulevard, Dallas, TX 75390

*To whom correspondence should be addressed. E-mail: Chuo.Chen@UTSouthwestern.edu

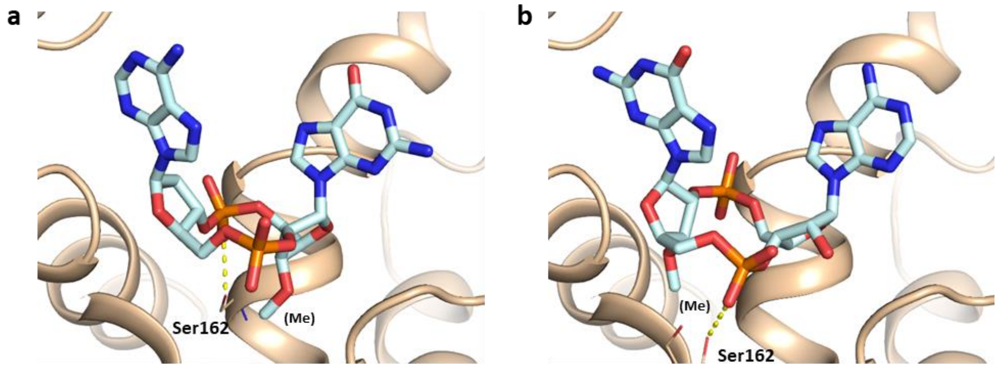

Figure S1. 3’*O*-Me-cGAMP (**1**) was modeled into the ligand-binding domain of STING (PDB: 4KSY) with same affinity as cGAMP. (a and b) The two orientations of **1** in complex with the symmetric STING dimer.

ligand	cGAMP	3'*O*-Me-cGAMP (**1**)	(R_P)-cGA^{S}MP (**2**)
STING dimer concentration (μM)	20	20	20
ligand concentration (μM)	200	280	200
Molar ratio (c-di-GMP:STING dimer, pre-bound)	3.5:1	3.5:1	3.5:1
K_d (nM)	8.62	2.69	13.72
68.3% confidence interval of K_d (nM)	[6.64, 11.23]	[1.66, 4.40]	[11.29, 17.15]
ΔH (kcal/M)	4.14	3.47	5.87
68.3% confidence interval of ΔH (kcal/M)	[3.86, 4.45]	[3.31, 3.63]	[5.73, 6.02]

Figure S2. Summary of the ligand-binding affinities of STING for different cGAMP analogs. Each ligand was titrated into apo-STING dimer and c-di-GMP-bound STING dimer (molar ratio of 3.5:1), respectively.

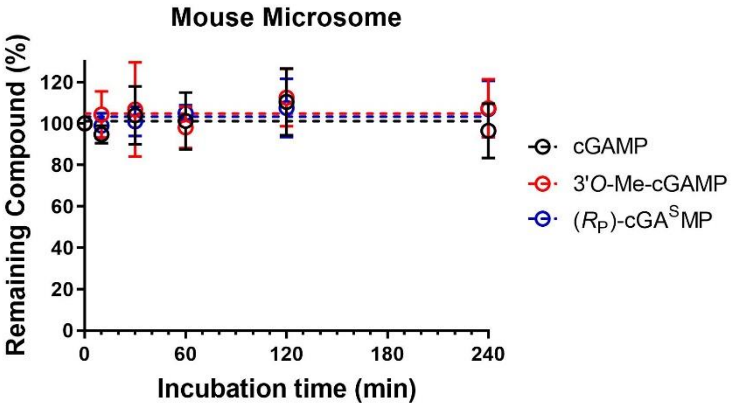

Figure S3. cGAMP, 3’*O*-Me-cGAMP (**1**) and (R_P)-cGA^{S}MP (**2**) remained intact after incubating with mouse liver microsomes for 2 h.

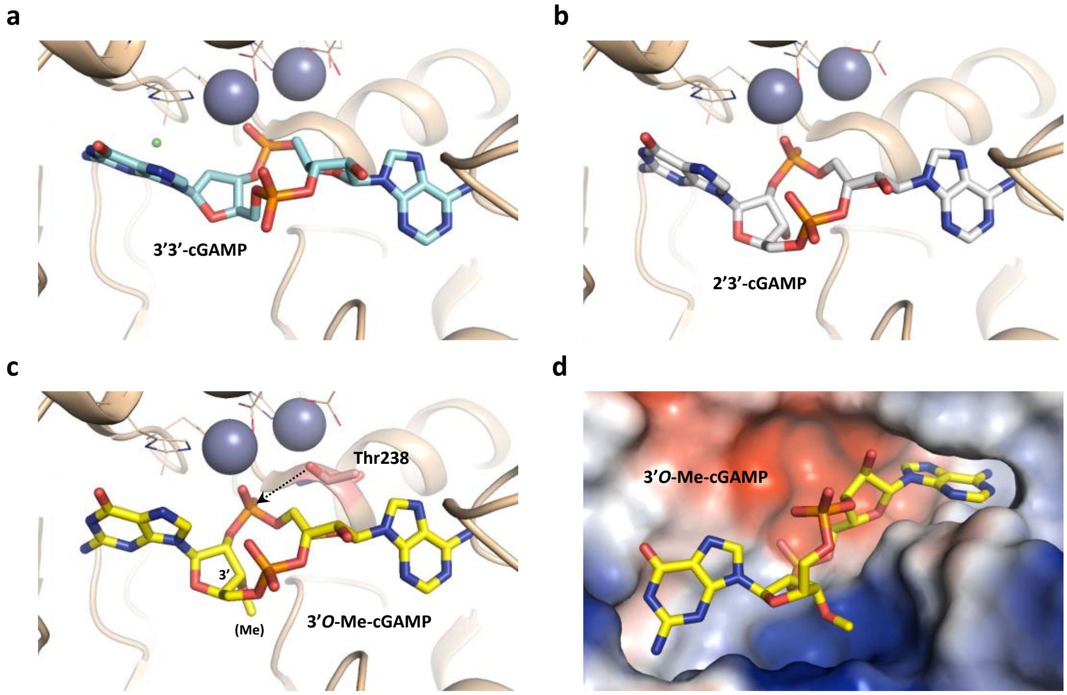

Figure S4. (a) Crystal structure (PDB 6AEL) of mouse ENPP1 in complex with 3’3’-cGAMP. (b) Crystal structure of (PDB 6AEK) of mouse ENPP1 (T238A) in complex with 2’3’-cGAMP (modeled). (c and d) Crystal structure of (PDB 6AEK) of mouse ENPP1 (with T238 modeled in) in complex with 3’*O*-Me-cGAMP (**1**) (modeled).

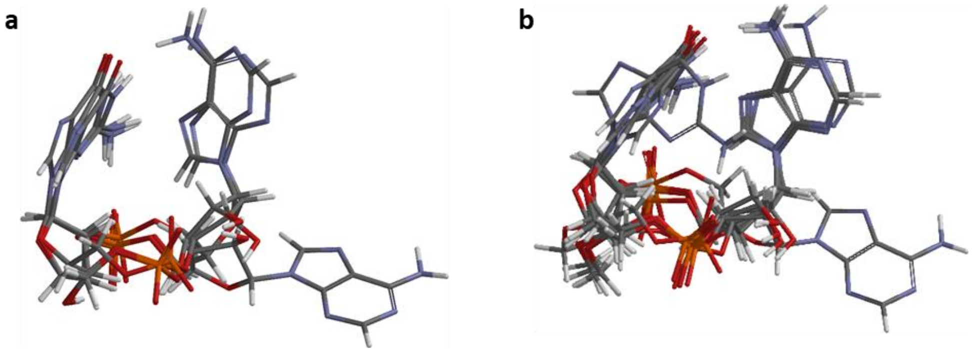

Figure S5. Conformation analysis identified the first open conformation of (a) cGAMP being 6.25 kcal/mol higher in energy than its lowest energy conformation, and that of (b) 3'*O*-Me-cGAMP (**1**) is 10.02 kcal/mol. The conformation search were performed with MMFF using the free ligand conformation[13] of cGAMP as the starting point. The single-point energy of the resulting conformers were subsequently assessed by DFT ωB97X-D/6-31G(d) with C-PCM water solvation using a dielectric constant of 78.30.

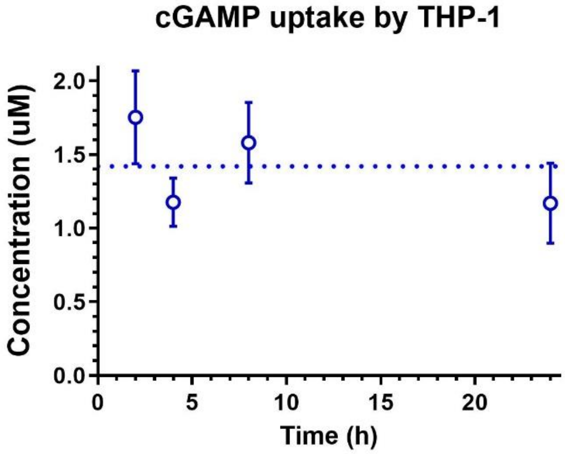

Figure S6. The intramolecular concentration of cGAMP was maintained at ~1.5 μM when incubating THP-1 cells with 10 μM cGAMP.

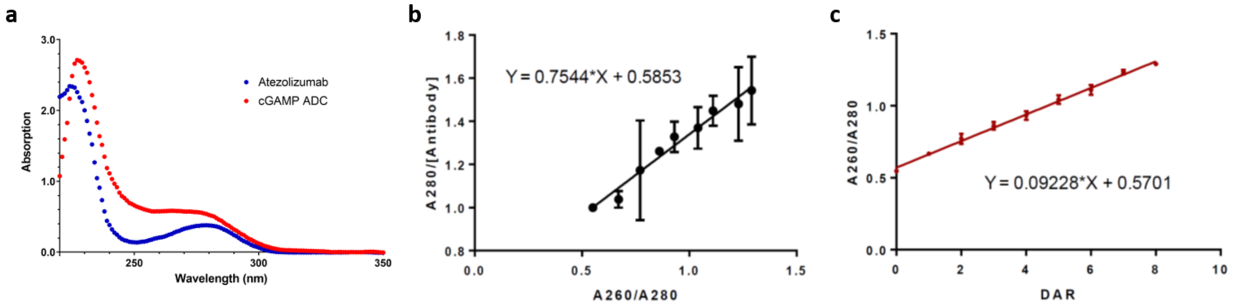

Figure S7. Calculation of the DAR by the UV profile of the ADC. (**a**) The UV spectra of ADC **8**/**8'**. (b) The calibration curve for the concentration of IgG. (c) The calibration curve for the DAR of the ADC.

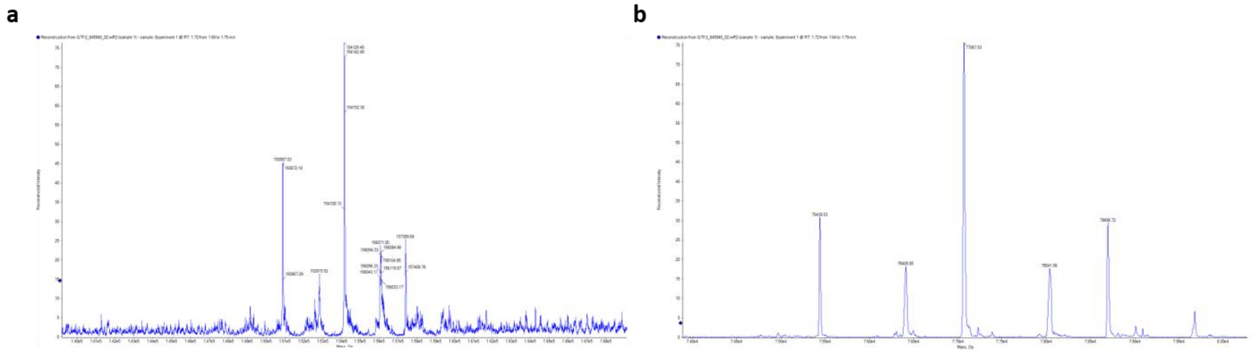

Figure S8. The deconvoluted intact mass spectra of ADC**8**/**8’** in the (a) 150 kDa region and (b) 75 kDa region.

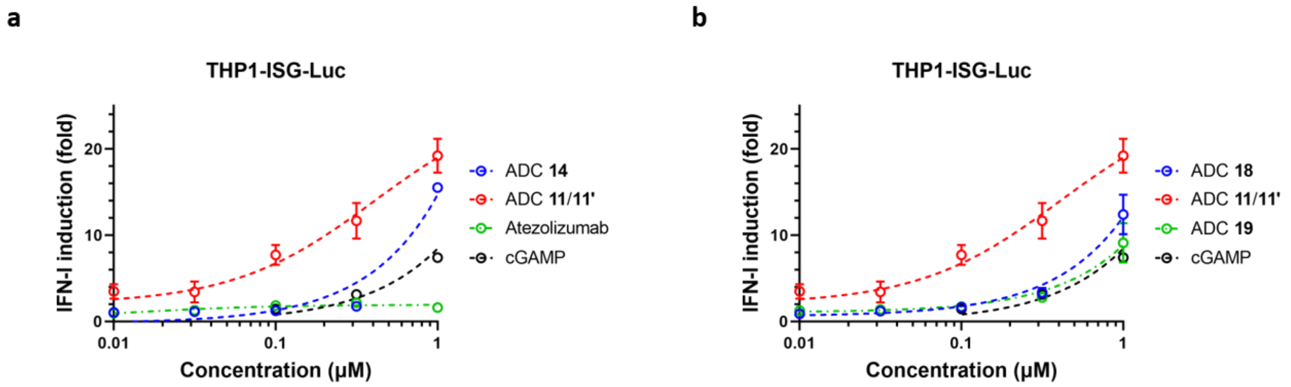

Figure S9. The activity of (a) ADC **14** and (b) ADC **18** and ADC **19** comparing to that of ADC **11/11’** in THP-1 cells.

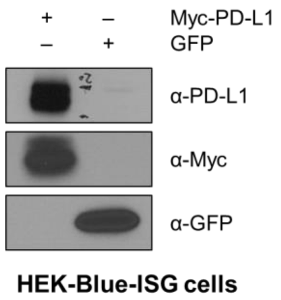

Figure S10. The Western blot analysis of HEK-Blue-ISG cells (Invivogen) transfected with Myc-PD-L1 or GFP (control) plasmid.

General Information

All solvents for the synthesis were purified by passing commercially available pre-dried, oxygen-free formulations through activated alumina columns. Reactions were monitored by TLC or LCMS and the products were purified by flash column chromatography unless otherwise mentioned. NMR spectra were recorded on a Bruker AN400 or AN600 instrument. The chemical shifts for ^{1}H and ^{13}C NMR spectra are reported in ppm (δ) relative to the ^{1}H and ^{13}C signals in the solvent (D_2O δ 4.79 ppm, $CDCl_3$ δ 7.26 ppm, methanol-d_4 δ 3.31 ppm) and the multiplicities are presented as follows: s = singlet, d = doublet, t = triplet, m = multiplet. LC-MS was performed on an Agilent 1260 HPLC machine coupled to a 6120 single quadrupole MS detector using an Agilent Eclipse XDB-C18 5 μm 4.6×150 mm column. **1** and **2** were synthesized using the previously reported methods (WO/2017/161349). The activity of the CDN and ADC is measured in THP1-Dual, Raw-Lucia-ISG, or HEK-Blue-ISG reporter cells purchased from Invivogen following protocols provided by the vendor. The ELISA kits for IFN-β (ProQuantum), CXCL10, TNF-α (ProQuantum) were purchased from ThermoFisher Scientific, IL-6 from BD Biosciences, and cGAMP from Cayman. The Myc-PD-L1 and GFP plasmids were obtained from Addgene.

General procedures for bis-sulfone conjugation. Azido-cGAMP (15 equiv) was incubated with bisulfone-DBCO (10 equiv) at 23 °C for 1 h to functionalize cGAMP with a linker for loading onto the antibody. Meanwhile, to a solution of atezolizumab (5 mg/mL, 1.0 equiv) in pH 7.4 PBS buffer containing 0.5 mM EDTA was added tris(2-carboxyethyl)phosphine hydrochloride (TCEP, 10 equiv). After incubating at 37 °C for 1 h, excess TCEP were removed by Vivaspin (MWCO 50 kD). The reduced atezolizumab was then diluted with PBS buffer to 0.5 mg/mL before the bis-sulfone-linker functionalized cGAMP (e.g., **6** or **10**) was added. After incubating at 23 °C for 24 h, excess conjugating reagents were removed by Vivaspin (MWCO 50 kD) and optionally purified by gel filtration using Sephadex G-50 to give the corresponding ADC.

General procedures for diBrPD conjugation. Azido-cGAMP (30 equiv) was incubated with diBrP-DBCO (20 equiv) at 23 °C for 1 h to functionalize cGAMP with a linker for loading onto the antibody. Meanwhile, to a solution of atezolizumab (5 mg/mL, 1.0 equiv) in pH 8.0 BBS buffer containing 0.5 mM EDTA was added TCEP (10 equiv) and the diBrPD-linker functionalized cGAMP (with 1% v/v DMSO). After incubating at 23 °C for 24 h, excess conjugating reagents were removed by Vivaspin (MWCO 50 kD) to give the corresponding ADC.

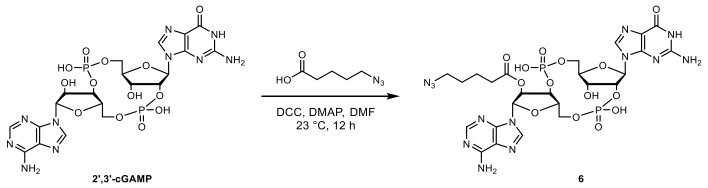

Synthesis of azido-C5-cGAMP (6). To a solution of 5-azidopentanoic acid (10.6 mg, 0.074 mmol, 10 equiv) in 0.5 mL of *N*,*N*-dimethylformamide was added dicyclohexylcarbodiimide (15.2 mg, 0.074 mmol, 10 equiv) and 4-dimethylaminopyridine (0.9 mg, 0.0074 mmol, 1.0 equiv) at 23 °C. After stirring for 15 min, 2’3’-cGAMP (5.0 mg, 0.0074 mmol, 1.0 equiv) was added and the reaction mixture was stirred for another 12 h before diluted with water, washed with ethyl acetate, and back extracted with water. The combined aqueous layers were then purified by preparation HPLC to give **6** (2.1 mg, 38% yield) as a white powder. ^{1}H NMR (500 MHz, D_2O) δ 8.26 (s, 1H), 8.20 (s, 1H), 7.86 (s, 1H), 6.21 (s, 1H), 5.93 (d, *J* = 8.5 Hz, 1H), 5.77 (d, *J* = 4.6 Hz, 1H), 5.65 (td, *J* = 8.2, 4.1 Hz, 1H), 5.21 (ddd, *J* = 9.1, 6.4, 4.6 Hz, 1H), 4.56 (d, *J* = 4.2 Hz, 1H), 4.52–4.46 (m, 1H), 4.43–4.32 (m, 2H), 4.23–4.07 (m, 3H), 3.33 (t, *J* = 6.7 Hz, 2H), 2.60 (q, *J* = 7.3 Hz, 2H), 1.73 (q, *J* = 7.4 Hz, 2H), 1.68–1.61 (m, 2H). MS (ESI) calcd for $C_{25}H_{32}N_{13}O_{14}P_2^+$ $(M+H)^+$ 800.2, found 800.1.

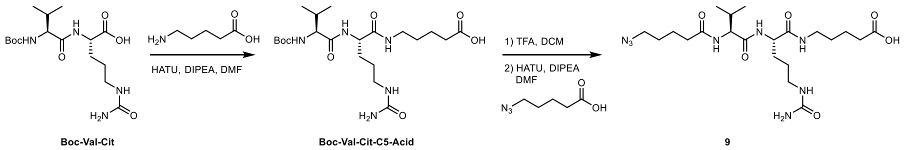

Synthesis of azido-C5-Val-Cit-C5-carboxylic acid (9). To a solution of Boc-Val-Cit (375 mg, 1.0 mmol, 1.0

equiv) in *N*,*N*-dimethylformamide (5 mL) was added diisopropylethylamine (345 µL, 2.0 mmol, 2.0 equiv) and HATU (380 mg, 1.0 mmol, 1.0 equiv) at 0 °C. After stirring for 40 min, 5-aminopentanoic acid (129 mg, 1.1 mmol, 1.1 equiv) was added and the reaction mixture was stirred at 23 °C for 4 h before purified by C18 reversed-phase flash column chromatography directly to give Boc-Val-Cit-C5-carboxylic acid (247 mg, 52% yield) as a white powder. MS (ESI) calcd for $C_{21}H_{40}N_5O_7^+$ $(M+H)^+$ 474.3, found 474.3.

To a solution of Boc-Val-Cit-C5-carboxylic acid (47 mg, 0.1 mmol) in methylene chloride (2 mL) was added trifluoroacetic acid (0.4 mL). After stirring at 40 °C for 3 h, the volatiles were removed to give Val-Cit-C5-carboxylic acid. MS (ESI) calcd for $C_{16}H_{32}N_5O_5^+$ $(M+H)^+$ 374.2, found 374.2.

To a solution of 5-azidopentanoic acid (15.7 mg, 0.11 mmol, 1.1 equiv) in *N*,*N*-dimethylformamide (0.5 mL) was added diisopropylethylamine (50 µL, 0.3 mmol, 3.0 equiv) and HATU (42 mg, 0.11 mmol, 1.1 equiv) at 0 °C. After stirring for 40 min, the Val-Cit-C5-carboxylic acid obtained above was added and the mixture was stirred at 23 °C overnight before purified by C18 reversed-phase flash column chromatography directly to give **9** (35 mg, 64% yield) as a white powder. ^{1}H NMR (400 MHz, D_2O) δ 4.29 (ddd, *J* = 11.2, 8.9, 5.6 Hz, 1H), 4.10 (dd, *J* = 19.4, 7.9 Hz, 1H), 3.39 (t, *J* = 6.6 Hz, 2H), 3.35–3.19 (m, 2H), 3.16 (td, *J* = 6.8, 1.4 Hz, 2H), 2.50–2.32 (m, 4H), 2.17–2.00 (m, 1H), 1.98–1.47 (m, 12H), 1.14–0.92 (m, 6H). MS (ESI) calcd for $C_{21}H_{39}N_8O_6^+$ $(M+H)^+$ 499.3, found 499.3.

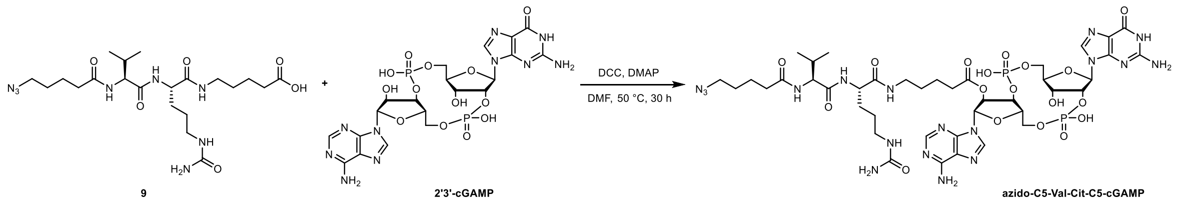

Synthesis of azido-C5-Val-Cit-C5-cGAMP. To a solution of 2’3’-cGAMP (11 mg, 0.015 mmol, 1.0 equiv) in *N*,*N*-dimethylformamide (0.5 mL) was added a mixture of **9** (7.4 mg, 0.015 mmol, 1.0 equiv), dicyclohexyl carbodiimide (61 mg, 0.3 mmol, 20 equiv) and 4-(dimethylamino)pyridine (2 mg, 0.015 mmol, 1.0 equiv) in *N*,*N*-dimethylformamide (0.5 mL) slowly over 30 h using a syringe pump. Upon completion, the reaction mixture was diluted with water, washed with ethyl acetate, and back extracted with water. The combined aqueous layers were then purified by preparation HPLC to give azido-C5-Val-Cit-C5-cGAMP (5.2 mg, 30% yield) as a white powder. ^{1}H NMR (600 MHz, D_2O) δ 8.30 (d, *J* = 5.2 Hz, 1H), 8.23 (s, 1H), 7.88 (s, 1H), 6.23 (s, 1H), 5.95 (d, *J* = 8.4 Hz, 1H), 5.80 (d, *J* = 4.5 Hz, 1H), 5.68 (s, 1H), 5.24 (s, 1H), 4.62–4.49 (m, 2H), 4.46–4.36 (m, 2H), 4.19 (ddd, *J* = 32.1, 25.0, 14.3 Hz, 6H), 4.08–3.97 (m, 2H), 3.32–3.03 (m, 16H), 2.89 (s, 1H), 2.59 (s, 2H), 2.31 (dq, *J* = 13.6, 6.9 Hz, 4H), 2.16 (t, *J* = 7.3 Hz, 1H), 2.00 (s, 2H), 1.70–1.40 (m, 9H), 1.10–0.82 (m, 6H). MS (ESI) calcd for $C_{41}H_{61}N_{18}O_{18}P_2^+$ $(M+H)^+$ 1155.4, found 1155.4.

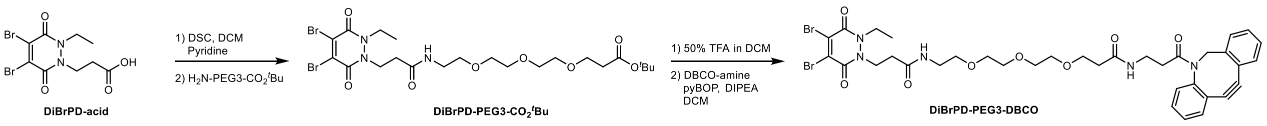

Synthesis of DiBrPD-PEG3-DBCO. To a solution of DiBrPD-acid (44 mg, 0.118 mmol, 1.0 equiv) in methylene chloride (1 mL) was added *N*-succinimidyl carbonate (45 mg, 0.177 mmol, 1.5 equiv) and pyridine (0.2 mL). After stirring at 55 °C for 2 h, the mixture was reaction concentrated and the residue was redissolved in acetonitrile (1 mL) before amino-PEG3-$CO_2{}^t$Bu (36 mg, 0.130 mmol, 1.1 equiv) was added. After stirring at 23 °C for 12 h, the reaction was quenched with water and extracted with ethyl acetate. The combined organic layers were washed with brine, dried over sodium sulfate, concentrated, and purified by silica gel flash column chromatography to give DiBrPD-PEG3-$CO_2{}^t$Bu (60 mg, 81% yield) as a white powder. ^{1}H NMR (400 MHz, $CDCl_3$) δ 6.82 (t, *J* = 5.5 Hz, 1H), 4.42–4.33 (m, 2H), 4.24 (qd, *J* = 7.0, 1.7 Hz, 2H), 3.66 (td, *J* = 6.4, 1.8 Hz, 2H), 3.57 (dd, *J* = 6.8, 2.1 Hz, 9H), 3.52–3.43 (m, 2H), 3.41–3.33 (m, 2H), 2.60–2.51 (m, 2H), 2.45 (td, *J* = 6.4, 1.8 Hz, 2H), 1.39 (d, *J* = 1.8 Hz, 9H), 1.23 (td, *J* = 7.1, 1.8 Hz, 3H). MS (ESI) calcd for $C_{22}H_{36}Br_2N_3O_8{}^+$ $(M+H)^+$ 628.1, found 628.1.

To a solution of DiBrPD- PEG3-$CO_2{}^t$Bu (60 mg, 0.096 mmol, 1.0 equiv) in methylene chloride (1 mL) was added trifluoroacetic acid (1 mL). After stirring at 40 °C for 3 h, the reaction mixture were concentrated and the residue was redissolved methylene chloride (1 mL) before PyBOP (55 mg, 0.106 mmol, 1.1 equiv) and diisopropylethylamine (20 µL, 0.106 mmol, 1.1 equiv) were added. After stirring at 23 °C for 30 min, DBCO-amine (29 mg, 0.105 mmol, 1.1 equiv) was added and the reaction mixture was stirred at 23 °C for 12 h before quenched with water and extracted with ethyl acetate. The combined organic layers were washed with brine, dried over sodium sulfate, concentrated, and purified by silica gel flash column chromatography to give DiBrPD-PEG3-DBCO (29.6 mg, 47% yield) as a brown powder. ^{1}H NMR (400 MHz, $CDCl_3$) δ 7.64 (dd, *J* = 7.4, 1.5 Hz, 1H), 7.47–7.22 (m, 7H), 7.11 (q, *J* = 4.3, 3.0 Hz, 1H), 6.85 (t, *J* = 6.1 Hz, 1H), 5.12 (d, *J* = 13.9 Hz, 1H), 4.47–4.16 (m, 7H), 3.77–3.21 (m, 17H), 2.63–2.42 (m, 3H), 2.35 (ddd, *J* = 6.5, 5.2, 3.3 Hz, 2H), 1.99 (ddd, *J* = 16.5, 6.9, 5.2 Hz, 1H), 1.26 (t, *J* = 7.1 Hz, 3H). MS (ESI) calcd for $C_{36}H_{42}Br_2N_5O_8{}^+$ $(M+H)^+$ 830.1, found 830.1.

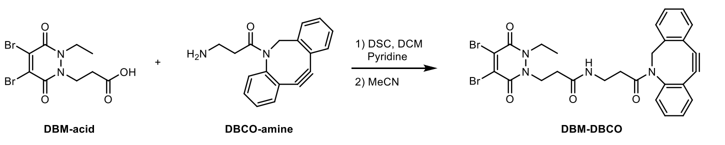

Synthesis of DiBrPD-DBCO. To a solution of DiBrPD-acid (30.5 mg, 0.082 mmol, 1.0 equiv) in methylene chloride (0.5 mL) was added *N*-succinimidyl carbonate (31.5 mg, 0.123 mmol, 1.5 equiv) and pyridine (0.1 mL). After stirring at 55 °C for 2 h, the reaction mixture was concentrated and the residue was redissolved in acetonitrile (0.5 mL) before DBCO-amine (22.7 mg, 0.082 mmol, 1.0 equiv) was added. After stirring at 23 °C for 12 h, the reaction was quenched with water and extracted with ethyl acetate. The combined organic layers were washed with brine, dried over sodium sulfate, concentrated, and purified by silica gel flash column chromatography to

give DiBrPD-DBCO (29 mg, 56% yield) as a brown powder. ^{1}H NMR (400 MHz, $CDCl_3$) δ 7.65 (dd, *J* = 7.6, 1.3 Hz, 1H), 7.44–7.35 (m, 5H), 7.32 (td, *J* = 7.5, 1.3 Hz, 1H), 7.31–7.21 (m, 2H), 7.17 (d, *J* = 11.2 Hz, 1H), 6.46 (t, *J* = 6.1 Hz, 1H), 5.14 (d, *J* = 14.0 Hz, 1H), 4.28 (ddtd, *J* = 34.2, 22.2, 15.0, 7.1 Hz, 4H), 3.71 (d, *J* = 13.9 Hz, 1H), 3.59 (s, 3H), 3.36 (dtd, *J* = 13.8, 7.0, 3.8 Hz, 1H), 3.13 (dddd, *J* = 13.5, 8.5, 5.4, 3.5 Hz, 1H), 2.48–2.32 (m, 4H), 2.03–1.92 (m, 1H), 1.25 (t, *J* = 7.1 Hz, 3H). MS (ESI) calcd for $C_{27}H_{25}Br_2N_4O_4^+$ $(M+H)^+$ 627.0, found 627.1.

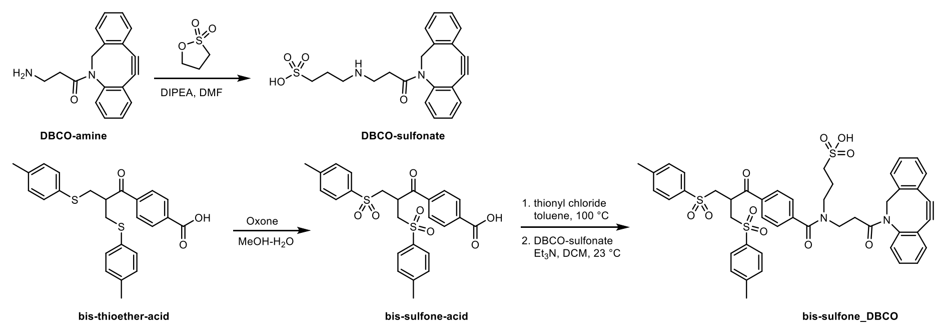

Synthesis of bis-sulfone-DBCO. To a solution of DBCO-amine (45.0 mg, 0.163 mmol, 1.0 equiv) in *N*,*N*-dimethylformamide (1 mL) was added 1,3-propanesultone (14.3 µL, 0.163 mmol, 1.0 equiv) and diisopropylethylamine (57.0 µL, 0.326 mmol, 2.0 equiv). After stirring at 23 °C for 12 h, the reaction mixture was concentrated and the residue was purified by silica gel flash column chromatography to give DBCO-sulfone (33.7 mg, 52% yield) as a white powder. MS (ESI) calcd for $C_{21}H_{23}N_2O_4S^+$ $(M+H)^+$ 399.1, found 399.1.

To a solution of compound bis-thioether-acid (87.2 mg, 0.2 mmol, 1.0 equiv) in a mixture of methanol (2 mL) and water (2 mL) was added Oxone (737.8 mg, 1.2 mmol, 6.0 equiv). After stirring at 23 °C for 12 h, the reaction was quenched with water and extracted with ethyl acetate. The combined organic layers were washed with brine, dried over sodium sulfate, concentrated, and purified by silica gel flash column chromatography to give bis-sulfone-acid (96.7 mg, 97% yield). MS (ESI) calcd for $C_{25}H_{25}O_7S_2^+$ $(M+H)^+$ 501.1, found 501.0.

To a solution of bis-sulfone-acid (19.6 mg, 0.039 mmol, 1.0 equiv) in anhydrous toluene (1 mL) was added thionyl chloride (6.2 µL, 0.078 mmol, 2.0 equiv). After stirring at 100 °C for 3 h, the reaction mixture was concentrated and the residue was redissolved in methylene chloride (1 mL) before DBCO-sulfone (17.2 mg, 0.043 mmol, 1.1 equiv) and triethylamine (27.3 µL, 0.196 mmol, 5.0 equiv) was added at 0 °C. After stirring at 23 °C for 5 h, the reaction mixture was concentrated and the residue was purified by silica gel flash column chromatography to give bis-sulfone-DBCO (21.2 mg, 61% yield). MS (ESI) calcd for $C_{46}H_{45}N_2O_{10}S_3^+$ $(M+H)^+$ 881.2, found 881.2.

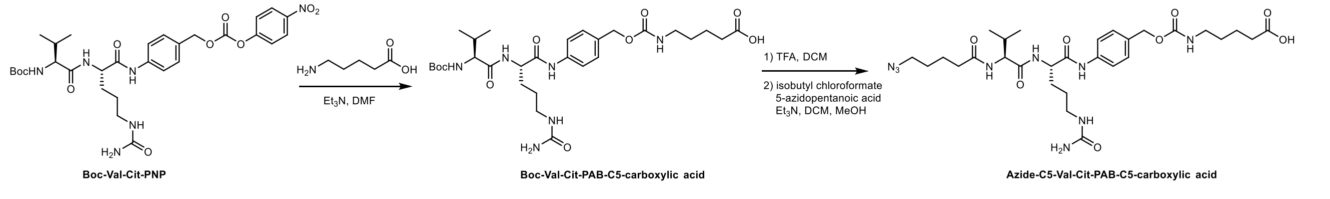

Synthesis of azido-C5-Val-Cit-PAB-C5-carboxylic acid. To a solution of Boc-Val-Cit-PNP (30.5 mg, 0.047

mmol, 1.0 equiv) in *N*,*N*-dimethylformamide (0.5 mL) was added 5-aminopentanoic acid (6.1 mg, 0.052 mmol, 1.1 equiv) followed by triethylamine (14.0 µL, 0.1 mmol). After stirring at 23 °C for 12 h, the reaction mixture was concentrated to give Boc-Val-Cit-PAB-C5-carboxylic acid. MS (ESI) calcd for $C_{29}H_{47}N_6O_9^+$ $(M+H)^+$ 623.3, found 623.4.

To a solution of the Boc-Val-Cit-PAB-C5-carboxylic acid obtained above in methylene chloride (1 mL) was added trifluoroacetic acid (0.2 mL). After stirring at 23 °C for 1 h, the reaction mixture was concentrated to give Val-Cit-PAB-C5-carboxylic acid. MS (ESI) calcd for $C_{24}H_{39}N_6O_7^+$ $(M+H)^+$ 523.3, found 523.3.

To a solution of 5-azidopentanoic acid (8.4 mg, 0.059 mmol, 1.26 equiv) in methylene chloride (0.6 mL) was added triethylamine (8.5 µL, 0.061 mmol, 1.3 equiv) followed by isobutyl chloroformate (7.3 µL, 0.056 mmol, 1.2 equiv) at 0 °C. After stirring for 30 min, the reaction mixture was added to a mixture of the Val-Cit-PAB-C5-carboxylic acid obtained above and triethylamine (8.5 µL, 0.061 mmol, 1.3 equiv) a mixture of methanol and methylene chloride (1:3 v/v, 1.2 mL). After stirring at 0 °C for 5 min and 23 °C for 2 h, the reaction mixture was concentrated and the residue was purified by silica gel flash column chromatography to give azido-C5-Val-Cit-PAB-C5-carboxylic acid (20.4 mg, 67% yield). ^{1}H NMR (400 MHz, methanol-d_4) δ 9.83 (s, 1H), 8.27 (d, *J* = 7.5 Hz, 1H), 8.01 (d, *J* = 7.8 Hz, 1H), 7.62–7.51 (m, 2H), 7.31 (d, *J* = 8.2 Hz, 2H), 5.02 (s, 2H), 4.51 (td, *J* = 8.2, 5.1 Hz, 1H), 4.18 (t, *J* = 7.7 Hz, 1H), 3.21 (p, *J* = 6.7, 6.1 Hz, 2H), 3.12 (t, *J* = 6.7 Hz, 3H), 2.31 (td, *J* = 7.3, 4.9 Hz, 4H), 2.08 (h, *J* = 6.9 Hz, 1H), 1.90 (td, *J* = 14.0, 12.8, 5.6 Hz, 1H), 1.81–1.25 (m, 12H), 0.98 (dd, *J* = 6.8, 4.4 Hz, 6H). MS (ESI) calcd for $C_{29}H_{45}N_9NaO_8^+$ $(M+Na)^+$ 670.3, found 670.3.

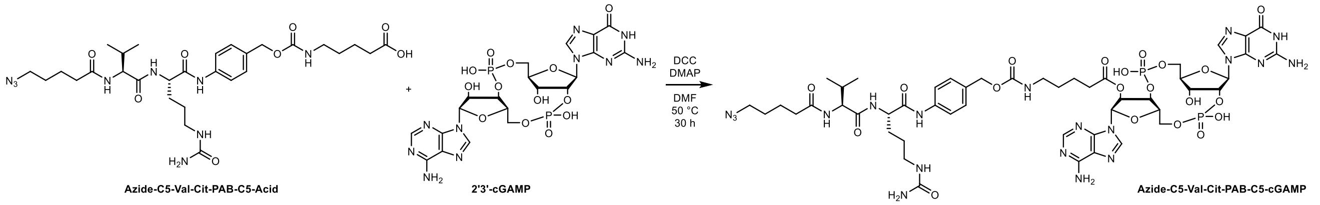

Synthesis of azido-C5-Val-Cit-PAB-C5-cGAMP. To a solution of 2'3'-cGAMP (4.4 mg, 0.0062 mmol, 1.0 equiv) in *N*,*N*-dimethylformamide (0.5 mL) was added a mixture of azido-C5-Val-Cit-PAB-C5-carboxylic acid (6.0 mg, 0.0093 mmol, 1.5 equiv), dicyclohexyl carbodiimide (12.7 mg, 0.062 mmol, 1.0 equiv) and 4-(dimethylamino)pyridine (3.8 mg, 0.031 mmol) in *N*,*N*-dimethylformamide (0.5 mL) over 30 h using a syringe pump. Upon completion, the reaction mixture was diluted with water, extracted with ethyl acetate, and back extracted with water. The combined aqueous layers were purified by preparation HPLC to give azido-C5-Val-Cit-PAB-C5-cGAMP (3.2 mg, 40% yield) as a white powder. MS (ESI) calcd for $C_{49}H_{68}N_{19}O_{20}P_2^+$ $(M+H)^+$ 1304.4, found 1304.4.

General procedure for DAR standard synthesis. To a solution of bis-sulfone-linker-DBCO or DiBrPD-linker-

DBCO (1.0 equiv) in dimethylsulfoxide was added the azido-acid (1.2 equiv) at 23 °C. After incubating for 2 h, 1-butanethiol (10 eq.) was added and the mixture was stirred at 23 °C for 12 h before purified directly by preparation HPLC to give the corresponding DAR standard for UV analysis.

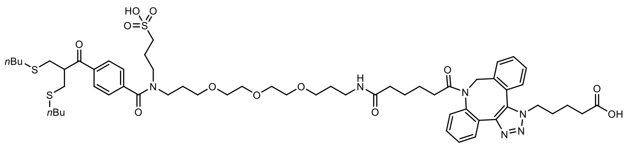

Bis-thioether-PEG3-DBCOTZ-C5-carboxylic acid

Bis-thioether-PEG3-DBCOTZ-C5-carboxylic acid. MS (ESI) calcd for $C_{58}H_{83}N_6O_{12}S_3^+$ $(M+H)^+$ 1151.5, found 1151.5.

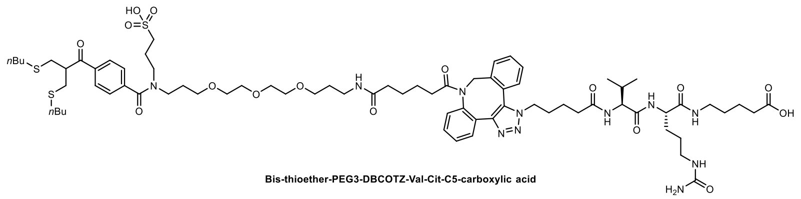

Bis-thioether-PEG3-DBCOTZ-Val-Cit-C5-carboxylic acid

Bis-thioether-PEG3-DBCOTZ-Val-Cit-C5-carboxylic acid. MS (ESI) calcd for $C_{74}H_{112}N_{11}O_{16}S_3^+$ $(M+H)^+$ 1506.7, $(M+2H^+)/2$ 753.9, found 753.9.

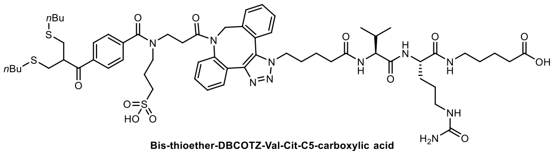

Bis-thioether-DBCOTZ-Val-Cit-C5-carboxylic acid

Bis-thioether-DBCOTZ-Val-Cit-C5-carboxylic acid. MS (ESI) calcd for $C_{61}H_{87}N_{10}O_{12}S_3^+$ $(M+H)^+$ 1247.6, found 1247.5.

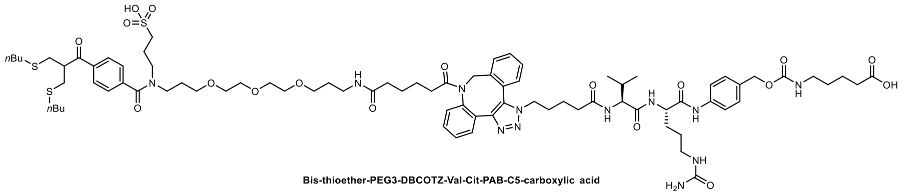

Bis-thioether-PEG3-DBCOTZ-Val-Cit-PAB-C5-carboxylic acid

Bis-thioether-PEG3-DBCOTZ-Val-Cit-PAB-C5-carboxylic acid. MS (ESI) calcd for $C_{82}H_{119}N_{12}O_{18}S_3^+$ $(M+H)^+$ 1655.8, $(M-C_6H_{11}NO_4+2H^+)/2$ 747.9, found 747.9.

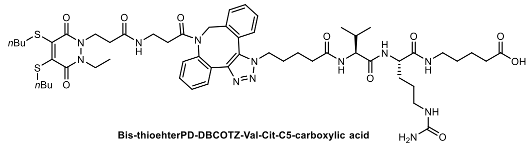

Bis-thioehterPD-DBCOTZ-Val-Cit-C5-carboxylic acid

Bis-thioehterPD-DBCOTZ-Val-Cit-C5-carboxylic acid. MS (ESI) calcd for $C_{56}H_{81}N_{12}O_{10}S_2^+$ $(M+H)^+$ 1145.6, found 1145.6.

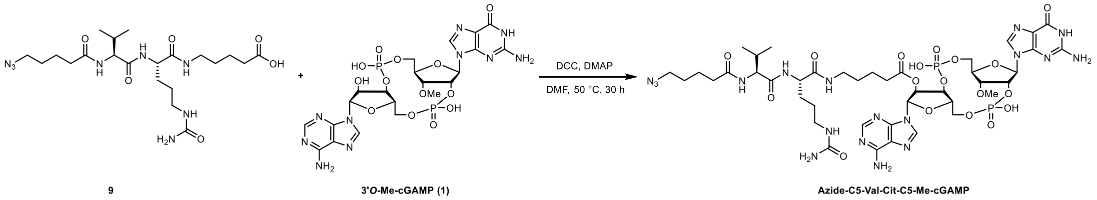

Synthesis of azido-C5-Val-Cit-C5-Me-cGAMP. To a solution of **1** (5.4 mg, 0.0078 mmol, 1.0 equiv) in *N*,*N*-dimethylformamide (0.5 mL) was added a mixture of **9** (5.9 mg, 0.012 mmol, 1.5 equiv), dicyclohexyl carbodiimide (16.2 mg, 0.078 mmol, 1.5 equiv), and 4-(dimethylamino)pyridine (4.8 mg, 0.039 mmol, 5.0 equiv) in *N*,*N*-dimethylformamide (0.5 mL) over 30 h using a syringe pump. Upon completion, the reaction mixture was diluted with water, extracted with ethyl acetate, and back extracted with water. The combined aqueous layers were purified by preparation HPLC to give azido-C5-Val-Cit-C5-Me-cGAMP (4.9 mg, 53% yield) as a white powder. MS (ESI) calcd for $C_{42}H_{63}N_{18}O_{18}P_2^+$ $(M+H)^+$ 1169.4, found 1169.4.

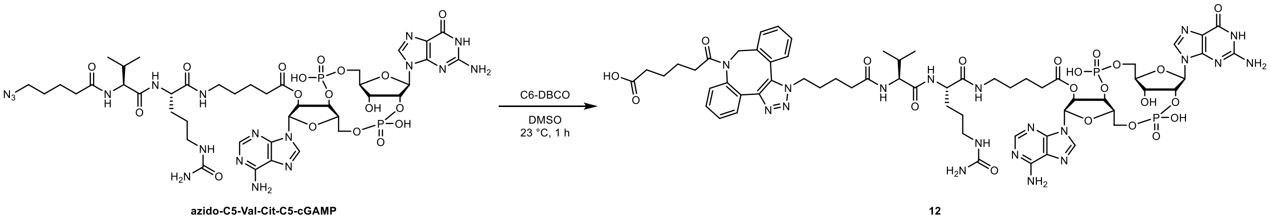

Synthesis of C6-DBCOTZ-Val-Cit-C5-carboxylic acid (12) To a solution of azido-C5-Val-Cit-C5-cGAMP (2.0 mg, 0.00173 mmol, 1.0 equiv) in dimethylsulfoxide (0.1 mL) was added C6-DBCO (0.6 mg, 0.00180 mmol). After stirring at 23 °C for 1 h, the reaction mixture was concentrated and the residue was purified by preparation HPLC to give **12** (2.2 mg, 85% yield) as a pale-yellow oil. MS (ESI) calcd for $C_{62}H_{80}N_{19}O_{21}P_2^+$ $(M+H)^+$ 1488.5, found 1488.2.

Cleavage of 12 with cathepsin B. A solution of DTT (0.3 M in water, 1 µL) and a solution of EDTA (0.15 M in water, 1 µL) were added to pH 5 sodium acetate buffer (100 mM, 98 µL) to obtain the activation solution (100 µL). Cathepsin B (22 µL, 0.1 U) was then added to the activation solution (10 µL) and incubated at 23 °C for 15 min before adding to a solution of **12** (10 mM in DMSO, 1 µL) in pH 5 sodium acetate buffer (100 mM, 467 µL). The amounts of **12** and cGAMP were then monitored by LC-MS.

Analytical LC-MS/MS conditions. Levels of cGAMP, 3'*O*-Me-cGAMP (**1**) and (R_P)-cGA^{S}MP (**2**) for *in vitro* and *in vivo* pharmacology assays were monitored by LC-MS/MS using an AB Sciex (Framingham, MA) 3200,

4000, 4500 or 6500+ QTRAP® mass spectrometer coupled to a Shimadzu (Columbia, MD) Prominence or Nexera X2 liquid chromatography system depending on the sensitivity needs of the experiment. For ADME assays the compounds were detected with the mass spectrometer in positive MRM (multiple reaction monitoring) mode by following the precursor to fragment ion transition 674.705 (m/z = 1) to 312.0 or 338.045 (m/z =2) to 136.0 (cGAMP); 688.808 (m/z = 1) to 312.0 or 344.981 (m/z = 2) to 152.1 (3'*O*-Me-cGAMP*)*; and 690.846 (m/z =1) to 136.0 or 346.014 (m/z = 2) to 152.0 ((R_P)-cGA^{S}MP). An Agilent C18 XDB column (5 micron, 50 X 4.6 mm) was used for chromatography of all three compounds with the following buffers: Buffer A: dH20 + 0.1% formic acid, Buffer B: acetonitrile + 0.1% formic acid. A flow rate of 1.5 ml/min was used for all, but gradient conditions varied. cGAMP: 0–0.5 min 0% B, 0.5–0.6 min gradient to 15% B, 0.6–1.6 min 15% B, 1.6–1.7 min gradient to 100%B, 1.7–4.6 min 100% B, 4.6–4.7min gradient to 0% B, 4.7–5 min 0% B, 3'*O*-Me-cGAMP: 0–0.5 min 0% B, 0.5–0.6 min gradient to 15% B, 0.6–1.6 min 15% B, 1.6–1.7 min gradient to 100% B, 1.7–4.6 min 100% B, 4.6–4.7 min gradient to 0% B, 4.7–5.0 min 0% B. (R_P)-cGA^{S}MP: 0–0.5 min 3% B, 0.5–0.6 min gradient to 20% B, 0.6–1.6 min 20% B, 1.6–1.7 min gradient to 100% B, 1.7–4.6 min 100% B, 4.6–4.7 min gradient to 3% B, 4.7–5.0 min 3% B. Tolbutamide (transition 271.2.2 to 91.2 from Sigma (St. Louis, MO) or *N*-benzylbenzamide (transition 212.1 to 91.1) also from Sigma was used as an internal standard (IS). A modified analytical assay with improved sensitivity was used to monitor release of cGAMP by enzymatic cleavage and in plasma from ADC constructs. The compound was again monitored in positive MRM mode but using an m/z = 2 charge for detection (transition 338.045/152.1). An ACUITY UPLC BEH Amide column (2.1x100 mm, 1.7 micron) was used with the following chromatography conditions: Buffer A: dH20 + 20 mM NH_4 acetate and Buffer B: 95% acetonitrile + 5% dH_20 + 2 mM NH_4 acetate + 0.1% acetic acid; flow rate of 0.15 ml/min and gradient conditions: 0–1.5 min 80% B, 1.5–6.0 min gradient to 30% B, 6.0–8.0 min 30% B, 8.0–9.0 min gradient to 80%B, 9.0–12.0 min 80%B; Flow rate: 0.15 mL/min. UMP-$^{13}C_9$ (transition 336.082 to 102.00) served as the internal standard.

Mouse liver microsome stability: Male ICR/CD-1 mouse microsome fractions (lot YMN) were purchased from Celsis/BioIVT (Baltimore, MD). Microsome protein (0.5 mg/mL) was placed in a glass screw cap tube; a 2 mM DMSO stock of each compound was spiked into a 50 mM Tris, pH 7.5 solution and this was added to the microsome solution on ice. The final concentration of compound after addition of all reagents was 2 µM. An NADPH-regenerating system (1.7 mg/ml NADP, 7.8 mg/ml glucose-6-phosphate, 6 U/ml glucose-6-phosphate dehydrogenase in 2% w/v $NaHCO_3$/10 mm MgCl2) was added for analysis of Phase I metabolism after heating both the regenerating solution and the sample tubes to 37 °C for 5 min in a 37 °C shaking water bath. The incubation was continued and at varying time points after addition of phase I cofactors, the reaction was stopped by the addition of 0.5 ml of methanol containing IS and formic acid such that the final concentration of IS was

50 ng/ml and acid was 0.1%. Time 0 samples were stopped with the methanol solution while still on ice prior to addition of the NADPH regenerating system and compound, which were subsequently added. The samples were incubated 10’ at RT and then spun at 16,100 × *g* for 5 min in a microcentrifuge at 4 °C. The supernatant was analyzed by LC-MS/MS and the percent of parent compound remaining at each timepoint was plotted. Metabolism of 7-ethoxycoumarin was used to monitor microsome performance. Assays were performed in triplicate for each compound.

Plasma Stability. CD1 mouse plasma isolated using acidified citrate dextrose (ACD) was purchased from BioIVT (Westbury, NY) and a DMSO stock of each compound was diluted into it at a final concentration of 2 µM. An aliquot was immediately removed for a zero time point and quenched with an equal volume of methanol containing 0.2% formic acid and 100 ng/ml Tolbutamide IS. The remainder of the sample was incubated in a 37 °C water bath for up to 24 hours. Samples were removed at the indicated times and processed as described. After vortexing and centrifugation to pellet protein, the supernatant was analyzed by LC-MS/MS as described above. A control incubation was conducted in saline (0.9% NaCl). Assays were performed in triplicate for each compound.

Hepatocyte Stability. Male ICR/CD-1 mouse hepatocytes, InVitroGRO HI and HT Medium, and Celsis Torpedo Antibiotic Mix were purchased from Celsis/BioIVT (Baltimore, MD). Cyropreserved hepatocytes were thawed in HT Media containing antibiotics, resuspended in HI media at 2 X 10^6/ml and plated in 96 well plates at 0.05 ml (10^5 cells)/well. Compounds to be tested were dissolved in DMSO at 2 mM and 0.05 ml added to the cells so that the final compound concentration was 2µM. Two additional wells containing compound and no cells were plated to serve as time 0 (C_0) and endpoint solvent control (C_{ep}). The cells were then placed in a 37 °C, 5% CO_2 incubator. Reactions were quenched with 200 µL of methanol containing tolbutamide IS and formic acid (final concentrations 50 ng/ml and 0.1%, respectively), vortexed for 15 seconds, incubated at room temperature for 10 minutes and spun at 4 °C for 5 mins at 16,100 × *g*. Supernatant was analyzed by LC-MS/MS using the conditions described above. The percent of parent compound remaining at each timepoint was plotted. Metabolism of 7-ethoxycoumarin was used to monitor hepatocyte performance. Assays were performed in triplicate for each compound.

Pharmacokinetic studies. Pharmacokinetic studies were performed by dosing 6 week old female CD1 mice (Charles River) with each compound by the subcutaneous route at 5 mg/kg. Compounds were dissolved in PBS and administered at 0.2 ml/animal. Animals were sacrificed in groups of three, blood was obtained by cardiac puncture at each time point (0, 10, 30, 60, 120, and 240 min post dose) using the anticoagulant ACD (acidified citrate dextrose) and plasma isolated by centrifugation. Plasma was mixed with a 2X volume of methanol

containing formic acid and internal standard. The samples were vortexed 15 sec, incubated at room temp for 10' and spun twice at 16,100 × *g* at 4 °C in a refrigerated microcentrifuge. The resulting supernatants were evaluated by LC-MS/MS as described above. Standard curves were generated using blank plasma (BioIVT, Westbury, NY) spiked with known concentrations of compound and processed as described above. The concentrations of drug in each time-point sample were quantified using Analyst software (Sciex, Redwood City, CA). A value of 3-fold above the signal obtained from blank plasma was designated the limit of detection (LOD). The limit of quantitation (LOQ) was defined as the lowest concentration at which back calculation yielded a concentration within 20% of theoretical). The cytokine levels were measured by the ELISA Kits following protocols provided by the vendors.

Excretion studies. Female CD1 mice of 6–10 weeks of age were dosed with compound as described above (5 mg/kg, 0.2 ml/mouse, subcutaneous route) in groups of three and placed in a metabolic cage (Tecniplast, Westchester, PA). Urine was collected from 0–4 hr, 4–8hr, and 8–24 hr and quantified as described above by LC-MS/MS. The concentration in urine was multiplied by the volume of urine collected and then divided by the absolute amount of compound administered to the three mice in the metabolic cage to calculate the % excreted in urine by each time point.

ADC stability studies. ADC **11/11’** (10 μL) was added to mouse plasma (40 μL) or PBS (40 μL) and incubated at 37 °C for 5 h before adding STF-1084 (2 mM in DMSO, 5 μL). After incubated at the same temperature for 2 h, DTT/EDTA (3 mM/1.5 mM, 10 μL) activated (15 min) cathepsin B (1 μL, Sigma-Aldrich) was added followed by acetate buffer (pH 5, 100 mM, 34 μL). The mixture was then incubated at 37 °C for 13 h before quenched with methanol (400 μL). After removing the precipitated proteins by centrifugation, an aliquot of the solution was diluted (100×) with the Immunoassay Buffer C and assayed by the 2’,3’-cGAMP ELISA kit to determine the amount of cGAMP.
